# Laccase Immobilization on Copper-Magnetic Nanoparticles for Efficient Bisphenol Degradation

**DOI:** 10.4014/jmb.2210.10032

**Published:** 2022-11-11

**Authors:** Sanjay K. S. Patel, Vipin C. Kalia, Jung-Kul Lee

**Affiliations:** Department of Chemical Engineering, Konkuk University, Seoul 05029, Republic of Korea

**Keywords:** Bisphenol A, copper-magnetic nanoparticle, covalent immobilization, laccase, reusability

## Abstract

Laccase activity is influenced by copper (Cu) as an inducer. In this study, laccase was immobilized on Cu and Cu-magnetic (Cu/Fe_2_O_4_) nanoparticles (NPs) to improve enzyme stability and potential applications. The Cu/Fe_2_O_4_ NPs functionally activated by 3-aminopropyltriethoxysilane and glutaraldehyde exhibited an immobilization yield and relative activity (RA) of 93.1 and 140%, respectively. Under optimized conditions, Cu/Fe_2_O_4_ NPs showed high loading of laccase up to 285 mg/g of support and maximum RA of 140% at a pH 5.0 after 24 h of incubation (4°C). Immobilized laccase, as Cu/Fe_2_O_4_-laccase, had a higher optimum pH (4.0) and temperature (45°C) than those of a free enzyme. The pH and temperature profiles were significantly improved through immobilization. Cu/Fe_2_O_4_-laccase exhibited 25-fold higher thermal stability at 65°C and retained residual activity of 91.8% after 10 cycles of reuse. The degradation of bisphenols was 3.9-fold higher with Cu/Fe_2_O_4_-laccase than that with the free enzyme. To the best of our knowledge, *Rhus vernicifera* laccase immobilization on Cu or Cu/Fe_2_O_4_ NPs has not yet been reported. This investigation revealed that laccase immobilization on Cu/Fe_2_O_4_ NPs is desirable for efficient enzyme loading and high relative activity, with remarkable bisphenol A degradation potential.

## Introduction

The industrial applications of enzymes are primarily limited by their low stability, substrate or solvent tolerance, and reusability. Various strategies, such as enzyme engineering and immobilization, have been adopted to improve stability [1, 2]. Despite the immense effort required in protein engineering, the results are undesirable and lead to only minor gains in enzyme stability [3]. Through immobilization, enzymes can enhance catalytic activity, stability, and reusability [4]. Several approaches for industrial enzyme immobilization have been reported, such as encapsulation within polymeric materials or metal–protein hybrids [5], adsorption on solid supports or membranes [6, 7], covalent immobilization on supports, biomolecules, or polymers [2, 8], and cross-linking mediated by linkers, such as glyoxal and glutaraldehyde [9, 10]. The additional treatment of glutaraldehyde on immobilized enzymes is beneficial to stabilize enzymes on solid supports, minimizing leaching and improving the structural stability of encapsulated enzymes [11]. Enzyme properties, such as residual activity, substrate specificity, and catalytic parameters, including turnover number, *V*_max_ and *K*_m_, pH, temperature profiles, stability (storage, room temperature, or thermal), and reusability highly vary among the different immobilization procedures [2, 12-14]. However, the selection of suitable approaches is essential for the successful immobilization of industrial enzymes. During adsorption, enzymes are attached to supports by weak bonds, such as van der Waals, hydrophobic, or ionic interactions [2]. In contrast, covalent procedures involve strong bonds, such as covalent interactions between support surfaces containing functional groups and various amino acids of the enzyme [15]. In addition, covalent methods more effectively minimize leaching, which is primarily associated with the adsorption of enzymes on the support surface [2, 16]. The support capacity for maximum enzyme loading is also a crucial parameter determining the effectiveness of immobilization procedures. The amount of enzyme immobilized is essentially altered by support assets, such as morphology, functional groups on their surface, surface area, and porosity, as well as enzyme properties, such as the size and number of groups involved in binding hydrophilic or hydrophobic behaviors [17, 18].

Nanoparticles (NPs) are considered practical supports for enzyme immobilization because of their unique assets: (i) commercial-scale availability, (ii) chemical alteration to provide suitable functional groups on the surface for enzyme binding, (iii) tunable to desirable sizes with high surface areas, modified to better biocompatibility, (iv) high rigidity to retain support stability during immobilization, and (v) magnetic nature for easy separation from the reaction mixture using a magnet over non-magnetic supports [2, 8, 12, 19, 20]. Numerous valuable enzymes, such as dehydrogenase, cellulase, laccase, and lipase, are widely used in industrial and environmental applications [3, 15, 21, 22]. Laccases are an oxidase group of enzymes that contain multi-copper (Cu). It is applied for purposes, such as the oxidation of harmful phenolic and non-phenolic compounds and degradation of pollutants or dyes [1, 23-26]. Bisphenols, such as bisphenol A and F, are extensively used to synthesize epoxy resins and polycarbonate plastics and are detectable in soft drinks and canned beverages or foods [26, 27]. These bisphenols exhibit high toxicity, cause oxidative stress, and have substantial endocrine-altering potential, especially reproductive and carcinogenic abilities [28, 29]. Therefore, degradation of bisphenols is required to minimize their harmful effects on aquatic and terrestrial organisms, including humans. Laccases can potentially degrade these bisphenols, but this is limited by the low degradation efficiency or stability of free enzyme forms [26, 29]. Various supports, including chitosan, sepiolite, silica, and magnetic NPs, were previously used for laccase immobilization [28-31]. Compared to other systems, the Cu metals-based encapsulation of laccases via metal-protein hybrids is beneficial to retain better residual activity due to the presence of Cu as an active center in laccase [16, 23, 32]. In addition, Cu-based hybrids also exhibit laccase-mimicking properties and are helpful in the degradation of phenolics [33, 34]. However, the Cu-containing magnetic NPs have never been used to immobilize laccase covalently. Therefore, in this study, laccase was immobilized on Cu and Cu-magnetic (Cu/Fe_2_O_4_) NPs functionally activated by 3-aminopropyltriethoxysilane (APTES) and glutaraldehyde to achieve better activity and easy separation.

## Materials and Methods

### Chemicals and Materials

APTES, 2,2'-azino-bis (3-ethylbenzothiazoline-6-sulfonic acid) (ABTS), Cu, Cu/Fe_2_O_4_ NPs, glutaraldehyde, and laccase were obtained from Sigma-Aldrich (USA). All other reagents and chemicals were of analytical grade.

### Functionalization of Nanoparticles and Laccase Immobilization

The NPs (100 mg) were activated with APTES (2%, v/v) in toluene by incubation for 12 h at room temperature (RT; 25°C) [12]. Glutaraldehyde (1 M) treatment after APTES was performed to provide aldehyde groups on the surface of the NPs at RT for 2 h in 100 mM phosphate buffer (pH 7) [30]. Initially, laccase was immobilized on various functionally activated NPs at pH 7.0 and incubated at 4°C for 24 h using 100 mg of protein/g for support. For efficient laccase immobilization on Cu/Fe_2_O_4_ NPs, the optimization parameters, including pH (4.0–8.0), temperature (4–16°C), the incubation period (6–32 h), and enzyme loading (50–600 mg of protein/g supports) were assessed. After immobilization, the NPs were separated by centrifugation (10,000 ×*g*, 15 min) and washed three times with phosphate buffer. The content of unbound enzymes in the supernatant was assessed by protein estimation [35]. The enzyme immobilization parameters were measured as follows (Eq. 1-2):

Immobilization yield (IY, %) = ratio of immobilized to added enzyme ×100, (1)

Relative activity (RA, %) = ratio of immobilized to free enzyme activity ×100, (2)

### Activity Assessment

Laccase activity was assessed spectrophotometrically at 420 nm (*ε*_max_ = 3.6 × 10^4^/M × cm) using ABTS [36].

### Immobilized Laccase Characterization and Kinetic Studies

The characteristics of Cu/Fe_2_O_4_-laccase were evaluated using the immobilized enzyme by field-emission scanning electron microscopy (FE-SEM) and Fourier transform infrared (FTIR) microscopy measurements [4, 23]. The decomposition of Cu/Fe_2_O_4_ and Cu/Fe_2_O_4_-laccase was compared to validate the high enzyme loading by thermogravimetric analysis (TGA) measurements [7]. The effect of pH on enzyme activity was evaluated in 100 mM of various buffers: glycine-HCl (2.5), sodium citrate (3.0–4.0), and sodium acetate (4.5–6.0). At optimum pH, the influence of temperature (25–70°C) on activity was compared between free and immobilized forms. The kinetic studies were performed using ABTS (0.05–20.0 mM) under standard assay conditions at 25°C in a 100 mM buffer. Furthermore, *V*_max_ and *K*_m_ values were derived using nonlinear regression fitting measurements (GraphPad Prism 5, USA) [14].

### Stability and Reusability Measurements

Initially, the storage stability of the enzyme at 4 and 25°C was evaluated for incubation for up to 30 d by measuring residual activity under standard assay conditions. Next, the thermal stability was assessed by incubating enzymes at various temperatures (40–70°C). The reusability of Cu/Fe_2_O_4_-laccase was assessed under standard assay conditions for up to 10 recycling cycles. After the first cycle, the Cu/Fe_2_O_4_-laccase was collected by centrifugation (10,000 ×*g*, 15 min) and used in the subsequent reaction. The zero- or initial-cycle activity of Cu/Fe_2_O_4_-laccase was measured as 100%.

### Bisphenols Degradation

Initially, the free and Cu/Fe_2_O_4_-laccase were assessed for their degradation ability of bisphenol A and F after 24 h of incubation using a concentration of 50 μM and 2 U/ml of enzyme at 25°C [36]. The residual bisphenol content was analyzed spectrophotometrically using a 4-AAP coupled reaction [35]. Furthermore, the degradation of bisphenols was evaluated at various concentrations (50–250 μM) after incubation for 12 h.

## Results and Discussion

### Laccase Immobilization on Cu/Fe_2_O_4_ NPs

**Fig. 1:**
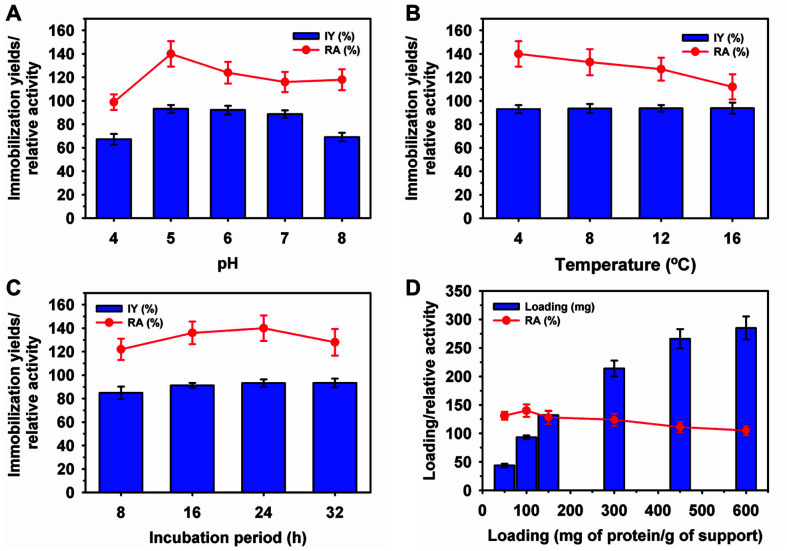
Influence of pH (A), temperature (B), incubation period (C), and loading of laccase on coppermagnetic nanoparticles. IY, Immobilization yield; RA, relative activity.

Free *R. vernicifera* laccase in the presence of Cu (5 mM) showed up to a 1.5-fold higher activity than laccase in the absence of Cu. Magnetic NP-immobilized enzymes could be easily separated from the reaction mixture by applying a magnetic field over non-magnetic supports. Therefore, laccase immobilization was demonstrated on pure Cu and Cu/Fe_2_O_4_ NPs functionally activated with glutaraldehyde, APTES, or APTES followed by glutaraldehyde. Initially, the IY and RA varied from 45.0 to 93.1% and 115 to 140%, respectively (Table 1). Based on the high IY and RA values, the Cu/Fe_2_O_4_ NPs were used for further investigation. Initially, immobilization was performed at a laccase loading of 100 mg/g of the support at various pH values (Fig. 1A). The IY and RA varied at 67.2–93.1 and 98.8–140%, respectively. The optimum pH was 5.0, with an IY of 93.1 and RA of 140%. This enhancement in RA correlated with the 50% higher activity in the presence of Cu metal ions (5 mM). Chitosan-based support-immobilized *R. vernicifera* laccase showed a very low IY of 0.4% [37] and RA of 36% [9]. After immobilization on a nylon membrane, *R. vernicifera* laccase showed a very low RA of approximately 3% compared to the free enzyme [6]. The incubation temperature had a variable influence on enzyme immobilization properties. The IY of laccase on Cu/Fe_2_O_4_ NPs was in the range of 93.1–93.8% at a temperature of 4–16°C (Fig. 1B). In contrast, the RA varied significantly between 140–112%. A lower RA at a higher temperature might be associated with an alteration in enzyme conformation during binding to the support [35]. The IY increased from 84.9 to 93.1% with an increase in incubation time from 8 to 24 h, and then stabilized at 93.3% (Fig. 1C). Previously, *R. vernicifera* laccase immobilized on sepiolite modified by Cu (Cu/sepiolite) or chitosan showed a low IY of up to 62% [31]. This finding suggested that Cu/Fe_2_O_4_ achieved a much higher IY of 93.3% compared to previous reports of laccase immobilization on Cu/sepiolite or chitosan [31, 37]. A slight decline in RA at higher incubation periods might be associated with the partial inactivation of the enzyme during immobilization [9, 38].

The loading of enzymes on a support is an essential parameter for assessing the success of immobilization [2]. The maximum laccase immobilization on Cu/Fe_2_O_4_ NPs was 285 mg/g of support at a loading rate of 600 mg of protein/g of support, which may be associated with the smaller size of NPs (Fig. 1D). Remarkably, at maximum loading, laccase exhibited a higher RA of 105% than the free form of the enzyme. Previously, a much lower loading of various laccases was reported on i) 14.2 mg/g of composite graphene oxide/CuFe_2_O_4_ composite NPs for *Trametes versicolor* [39], ii) 32.3 mg/g of zirconium chloride NPs for *R. vernicifera* [40], iii) up to 82.6 mg/g of magnetic carbon chemicals NPs for *Bacillus subtilis* [29], and iv) a maximum of 120 mg/g of Fe_3_O_4_@MoS_2_ core-shell NPs modified by polyethyleneimine for an unknown source of laccase [27].

### Characterization of Immobilized Laccase

**Fig. 2:**
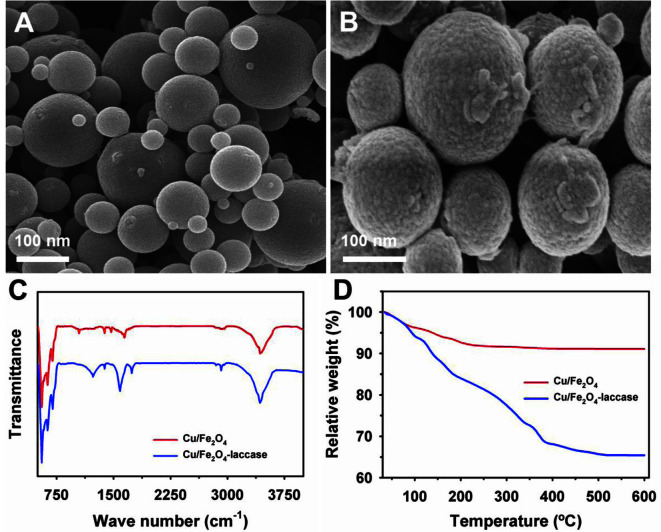
Characterization of laccase immobilized on copper-magnetic (Cu/Fe_2_O_4_) nanoparticles (NPs): Field emission scanning electron microscopy images of pure NPs (A), and immobilized laccase (B), Fourier transform infrared microscopy (C), and thermogravimetric (D) analysis.

FE-SEM analysis confirmed efficient laccase binding by the thick, rough texture of the NP surfaces after immobilization (Figs. 2 A and 2B). Furthermore, laccase immobilization on Cu/Fe_2_O_4_ NPs was validated by FTIR analysis in the wavenumber ranges of 500–4,000 cm^-1^ (Fig. 2C). The peaks between 550 and 600 cm^-1^ vibrations were associated with Fe-O. In addition, the peaks at 1385 and 1590 cm^-1^ peaks corresponded to antisymmetric and symmetric carboxylic group stretching, respectively. The broad peaks at 1050, 1230, and 3425 cm^-1^ represented the stretching vibrations of the alkoxy, epoxy, and hydroxyl groups, respectively. Comprehensive C=O stretching peaks at 1,650 cm^-1^ (amide I band) and N−H bending at 1,550 (amide II band) vibrations noted in the range of 1,550–1,650 cm^-1^ validated the immobilization of laccase on the Cu/Fe_2_O_4_ NPs. Effective laccase immobilization on Cu/Fe_2_O_4_ was assessed by TGA (Fig. 2D). Pure Cu/Fe_2_O_4_ exhibited a nearly 8.9% reduction in weight loss at 600°C. Under similar conditions, a high weight reduction of 34.6% for Cu/Fe_2_O_4_-laccase validated a maximum NP loading of 285 mg/g.

**Fig. 3:**
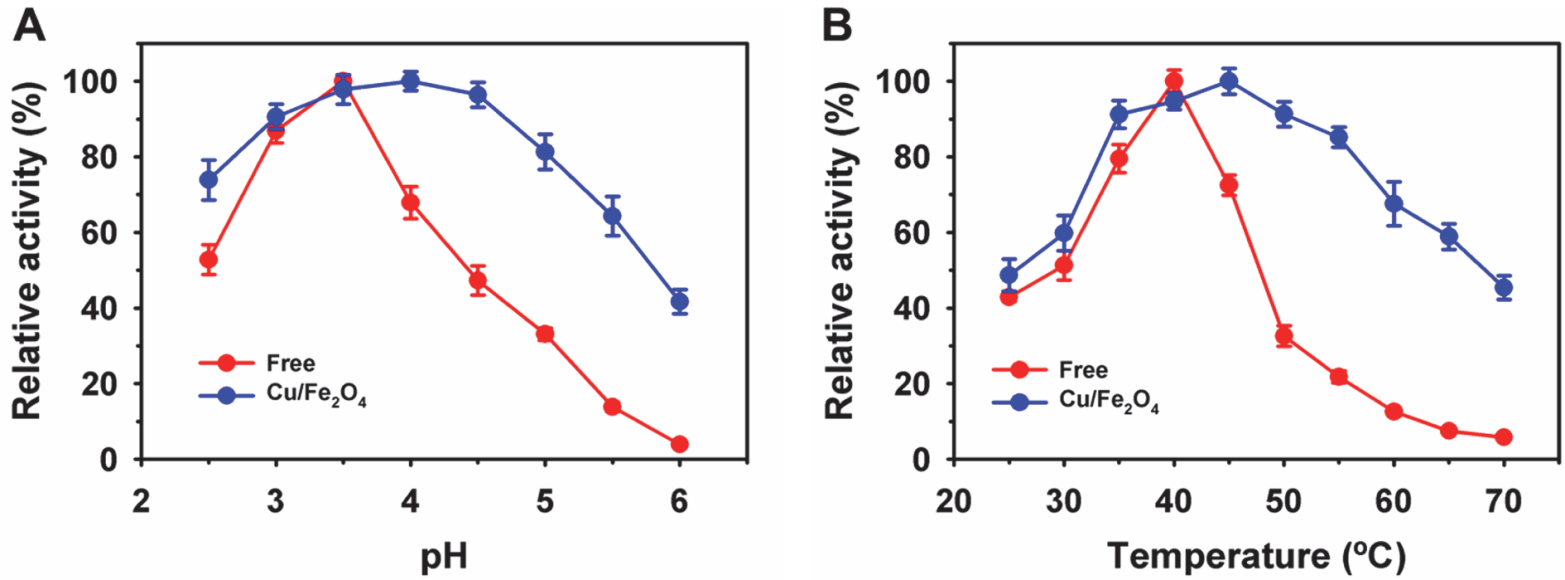
Laccase immobilized on copper-magnetic nanoparticles activity profiles at various pH (A), and temperature (B).

Enzyme properties, such as the pH and temperature profile, are significantly altered after immobilization [37]. Initially, free and Cu/Fe_2_O_4_-laccase activities were compared at a pH range of 2.5–6.0 (Fig. 3A). The free laccase exhibited an optimum pH of 3.5, with residual activity of 52.8% at pH 2.5 and 3.9% at pH 6.0. Immobilized laccase on Cu/Fe_2_O_4_ showed a higher optimum pH of 4.0 and higher retention of residual acidity of 1.4- and 10.7-fold at pH 2.5 and 6.0, respectively, than the free enzyme. The optimum pH for laccase was highly variable, depending on the type of substrates, such as ABTS, 2,6-dimethoxyphenol, and 4-phenylenediamine [12, 30, 37]. Previously, no shift in optimum pH was reported for *R. vernicifera* laccase immobilized on: i) 7.0 and 7.5 toward 4-phenylenediamine on chitosan [37], and zirconium chloride NPs [40], respectively; ii) 7.5 toward quinone on nylon membranes [6]; and iii) 7.5 for phenol on polypropylene membranes [41]. Free and Cu/Fe_2_O_4_-laccase showed optimum temperatures of 40 and 45°C, respectively (Fig. 3B). Previously, free and immobilized *R. vernicifera* laccase on zirconium chloride and chitosan NPs showed similar optimal temperatures of 40 and 45°C, respectively [9, 40]. A similar enhancement in optimal temperature was reported after the immobilization of *B. subtilis* laccase on magnetic carbon chemicals [29]. Free laccase exhibited a significant decline in residual activity at higher temperatures above 40°C. The residual activity was reduced by 87.4 and 94.2% at 60 and 70°C, respectively. Under similar conditions, Cu/Fe_2_O_4_-laccase maintained high residual activities of 67.6% at 60°C and 45.4% at 70°C. After immobilization on supports, a shift in the pH and temperature profiles of laccase might be linked to its effective binding to supports, which can result in suitable enzyme structural alteration or stabilization.

The kinetic parameters *K*_m_ of 1.72 mM and *V*_max_ of 68.3 μmol/min/mg of protein were determined using ABTS as a substrate for free laccase (Table 2). After immobilization on Cu/Fe_2_O_4_, laccase showed better affinity toward ABTS, which was evidenced by a lower *K*_m_ value of 1.23 mM and *V*_max_ of 95.6 μmol/min/mg of protein. Previously, laccase covalently immobilized on magnetic NPs exhibited a higher *K*_m_ than free enzymes because of the strong binding of the enzyme to the support, resulting in substrate transfer limitations or undesirable conformation alterations [30, 39]. An enhancement of 1.4-fold in *V*_max_ was noted for Cu/Fe_2_O_4_-laccase toward ABTS. Previously, GO-CuFe_2_O_4_-based *T. versicolor* laccase showed a significant decline of 2.2-fold in *V*_max_ of 26 mM/min over the free enzyme (56 mM/min) [39]. Nylon membrane-immobilized *R. vernicifera* laccase also exhibited a significant drop of 35-fold in *V*_max_ toward quinone compared to the maximum *V*_max_ of 9.58 μmol/min for free enzymes [6]. Overall, the enhancement in *K*_m_ or decline in *V*_max_ of laccase could be linked to substrate diffusion limitation or alteration in enzyme conformation by robust interactions of the enzyme with immobilizing supports. Compared to various other supports, Cu/Fe_2_O_4_ proved beneficial for efficient laccase immobilization with better kinetic parameters (Table 3). The enzyme immobilization on Cu/Fe_2_O_4_ helps improve *K*_m_ and *V*_max_ by the dependence of laccase on Cu as a catalytic inducer.

### Stability and Reusability

**Fig. 4:**
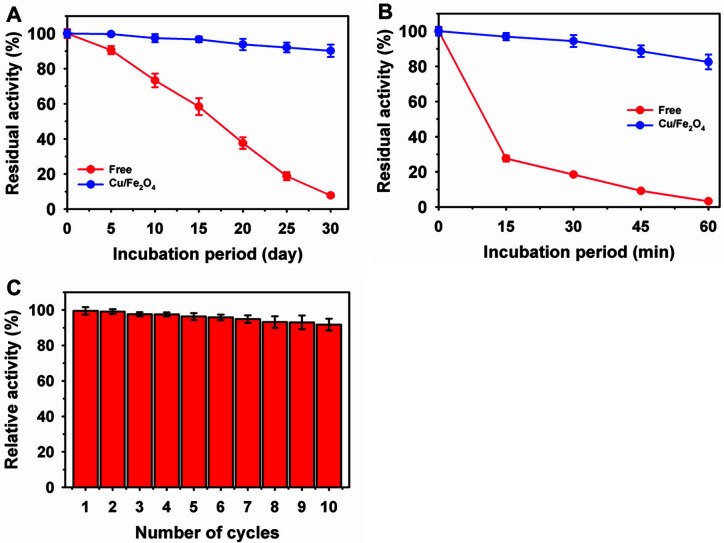
Stability measurement of laccase immobilized on copper-magnetic nanoparticles at 4°C (A), thermostability at 60°C (B), and reusability (C).

The principal objective of immobilization is to achieve improved enzyme stability [10, 13]. The strength of enzymes is highly altered, primarily depending on the immobilization procedures or support properties, such as surface area, morphology, and porosity. The thermostabilities of free and Cu/Fe_2_O_4_-laccase were compared at 60°C (Fig. 4A). Free laccase exhibited a progressive decline in residual activity over an incubation period of up to 60 min, and a 96.7% reduction in the residual activity was observed. Under similar conditions, Cu/Fe_2_O_4_-laccase retained a high residual activity of 73.4%, and the thermostability of the immobilized laccase was enhanced by up to 25-fold. Previously, a lower improvement of up to 3-fold in thermostability at 60°C was reported after immobilization of various laccases from *T. versicolar* on GO-based composites [39], *B. subtilis* on magnetic carbon chemicals [29], *R. vernicifera* on chromic acid modified polypropylene membranes [41], and *Trametes maxima* on amino-modified chicken feather-derived NPs [13]. Furthermore, the storage stability of free and immobilized laccase was evaluated at 4°C after incubation for 30 d (Fig. 4B). At the end of the incubation, the free laccase lost 92.8% of its residual activity, whereas the Cu/Fe_2_O_4_-laccase retained a much higher residual activity (92.2%). Previously, the relatively similar storage stability of free and immobilized laccase was reported on chicken feather NPs after incubation for 21 d at 4°C [13]. Overall, an 11.6-fold higher storage stability was achieved for Cu/Fe_2_O_4_-laccase, which may be correlated with the better structural stability of pure NPs over biodegradable supports. In contrast, only 2-fold storage stability was improved for laccase immobilized on GO-based composites over 30 d [39].

The immobilization of enzymes on magnetic supports is beneficial for their easy separation in the presence of a magnetic field over nonmagnetic supports [36, 39, 42]. Here, the reusability of Cu/Fe_2_O_4_-laccase was assessed under standard assay conditions using ABTS for up to 10 cycles (Fig. 4C). Cu/Fe_2_O_4_-laccase showed a minor, gradual decline in residual activity after consecutive operations, which might be associated with the leaching of NPs or partial inactivation of the enzyme during recycling. Moreover, Cu/Fe_2_O_4_-laccase retained much higher residual activity (96.3% after five cycles and 91.8% after 10 cycles of reuse), which can be associated with the supportive role of Cu in NPs on laccase activity. After 10 cycles of reusability, various immobilized laccases had lower residual activity: i) 21% for *T. versicolor* laccase on pure magnetic NPs, ii) up to 40% for *T. versicolor* laccase on various supports of poly(amidoisophthalic acid), cyclodextrin-anchored, or chitosan-coated magnetic NPs [28]; iii) 62% for an unknown source of laccase on polyethyleneimine-modified Fe_3_O_4_@MoS_2_ core-shell NPs [27]; and iv) 80% for *R. vernicifera* laccase on chitosan [9].

### Degradation of Bisphenols

**Fig. 5:**
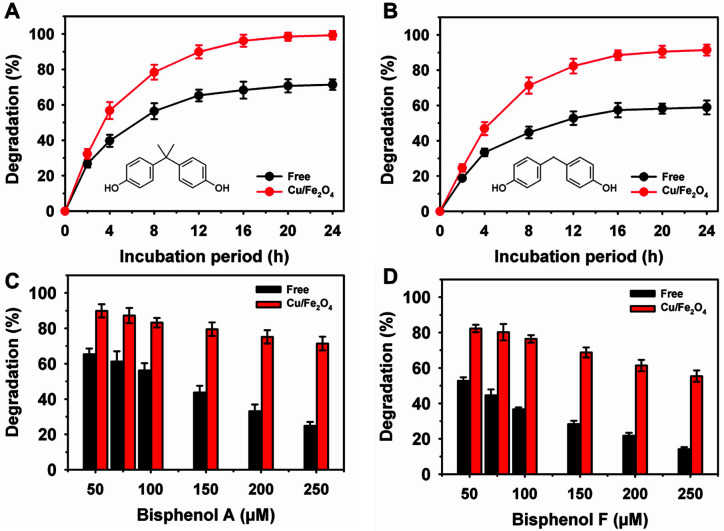
Bisphenol A degradation by laccase immobilized on copper-magnetic nanoparticles profile (A), various concentrations (B), and reusability (C). The degradation of bisphenols was performed by free and immobilized enzymes at 25°C.

The degradation of bisphenols F and A by free laccase was 58.9 and 71.4%, respectively, after 24 h of incubation with an initial bisphenol concentration of 50 μM (Figs. 5A and 5B). Under similar conditions, Cu/Fe_2_O_4_-laccase showed better degradation of 91.4% for bisphenol F and 99.3% for bisphenol A. A lower maximum bisphenol A degradation of up to 68% was observed for laccase immobilized on poly(amidoisophthalic acid)- and cyclodextrin-anchored-based Fe_3_O_4_ NPs [28]. Furthermore, the degradation of these bisphenols was evaluated at higher concentrations of up to 250 μM (Figs. 5C and 5D). The degradation of these bisphenols declined at increased concentrations (250 μM) by free and Cu/Fe_2_O_4_-laccase. At a bisphenol concentration of 250 μM, a maximum degradation of 14.2 and 24.9% by free laccase was achieved for bisphenol F and bisphenol A, respectively. In contrast, Cu/Fe_2_O_4_-laccase exhibited a much higher maximum degradation of 55.4% for bisphenol F and 71.4% for bisphenol A. After immobilization, laccase on Cu/Fe_2_O_4_ NPs showed 2.9- and 3.9-fold improvement in the degradation of bisphenol A and bisphenol F, respectively. Previously, bacterial laccase immobilized on magnetic carbon NPs showed a lower improvement (1.5-fold) in bisphenol A degradation [29]. Similarly, an enhancement in the degradation of only 3% for bisphenol A and 1.5-fold for bisphenol F was reported for laccase immobilized on polyethyleneimine-modified Fe_3_O_4_@MoS_2_ core-shell NPs [27].

Thus, here laccase was immobilized on Cu/Fe_2_O_4_ NPs to improve its residual activity, stability, and potential biotechnological applications. After immobilization on Cu/Fe_2_O_4_ NPs functionalized by APTES followed by glutaraldehyde, laccase exhibited a higher IY and better RA than the free enzyme. The immobilized laccase showed much better activity profiles over broad pH and temperature ranges. Moreover, a significant increase in the stability of laccase, with high reusability after 10 cycles of reuse, was achieved after immobilization. Cu/Fe_2_O_4_-laccase showed significantly higher degradation of bisphenols than the free enzyme. To the best of our knowledge, this is the first report of laccase (*R. vernicifera*) immobilization on pure Cu or Cu/Fe_2_O_4_ NPs. These findings demonstrated that laccase immobilization on Cu-based magnetic NPs effectively achieves high activity, stability, reusability, easy separation, and biotechnological applications.

## Figures and Tables

**Table 1:** Laccase immobilization on copper (Cu) and Cu-magnetic (Cu/Fe_2_O_4_) nanoparticles (NPs).

NPs	Functional activation	Immobilization yield (%)	Relative activity (%)
Cu/Fe_2_O_4_	Glutaraldehyde	82.5 ± 4.6	121 ± 10.2
	APTES	51.2 ± 4.2	115 ± 8.9
	APTES + glutaraldehyde	93.1 ± 3.3	140 ± 10.8
Cu	Glutaraldehyde	78.4 ± 4.8	125 ± 10.6
	APTES	45.0 ± 4.1	122 ± 9.5
	APTES + glutaraldehyde	90.2 ± 3.9	136 ± 11.3

The enzyme immobilization was performed in phosphate buffer (100 mM, pH 7) with a loading of 100 mg of protein/g of support for incubation of 24 h at 4°C.

**Table 2:** Kinetic parameters of free and immobilized enzyme on Cu/Fe_2_O_4_ NPs.

Laccase	*K*_m_ (mM)	*V*_max_ (μmol/min/mg protein)
Free enzyme	1.72 ± 0.38	68.3 ± 6.2
Immobilized enzyme	1.23 ± 0.31	95.6 ± 8.7

The kinetics parameters were measured using ABTS (0.05–20.0 mM) at 25°C in sodium citrate buffer (100 mM) at optimum pH of free and immobilized laccase.

**Table 3:** Immobilization and kinetic parameters of laccases on various supports.

Supports	Laccase source	Immobilization yield (%)	Relative activity (%)	*K*_m_ (mM)	*V*_max_ (μmol/min/mg protein)	Reference
Cu-alginate	*Trametes versicolor*	-^a^	88.8	0.56 (2.21)^c^	44.6 (5.4)	[16]
Chitosan	*Rhus vernicifera*	56.0	30.0	-	-	[9]
Graphene oxide/ CuFe_2_O_4_	*T. versicolor*	14.2^b^	88.0	1.80 (1.30)	26.0 (56.0) mM/min	[39]
Nylon membrane	*R. vernicifera*	-	2.80	11.3 (69.0)	0.27 (9.58)	[6]
Silica	*T. versicolor*	75.8	92.9	0.046 (0.029)	1630 (1890)	[30]
Titania	*Pleurotus ostreatus*	0.80^b^	126	0.043 (0.037)	101 (75.5)	[19]
Fe_3_O_4_@MoS_2_	-^a^	4.70	80.0	58.4 (58.1)	30.3 (31.8) mM/min	[27]
Fe_3_O_4_	*T. versicolor*	49.0	45.3	0.065 (0.029)	1140 (1890)	[7]
Cu/Fe_2_O_4_	*R. vernicifera*	93.8	140	1.23 (1.72)	95.6 (68.3)	This study

^a^Not available or applicable; ^b^amount of enzyme immobilized in mg/g of support; ^c^values within parenthesis are of free enzyme.
